# Robotic adrenalectomy in the pediatric population: initial experience case series from a tertiary center

**DOI:** 10.1186/s12894-020-00727-x

**Published:** 2020-10-07

**Authors:** Anirban P. Mitra, Evalynn Vasquez, Paul Kokorowski, Andy Y. Chang

**Affiliations:** 1 Institute of Urology, University of Southern California, Los Angeles, CA USA; 2 Division of Urology, Children’s Hospital Los Angeles, Los Angeles, CA USA

**Keywords:** Minimally-invasive surgery, Robotics, Pediatrics, Adrenal mass, Neuroblastoma, Pheochromocytoma, Case report

## Abstract

**Background:**

Laparoscopic resection is the most well described minimally-invasive approach for adrenalectomy. While it allows for improved cosmesis, faster recovery and decreased length of hospital stay compared with the open approach, instrument articulation limitations can hamper surgical dexterity in pediatric patients. Use of robotic assistance can greatly enhance operative field visualization and instrument control, and is in the early stages of adoption in academic centers for pediatric populations.

**Case presentation:**

We present a single-institution series of pediatric adrenalectomy cases. The da Vinci Xi surgical system was used to perform adrenalectomies on three consecutive patients (ages, 2–13 years) at our center. Final pathology revealed ganglioneuroblastoma (n = 2) and pheochromocytoma (n = 1). Median operating time was 244 min (range, 244–265 min); median blood loss was estimated at 100 ml (range, 15–175 ml). Specimens were delivered intact and all margins were negative. Median post-operative hospital stay was 2 days (range, 1–6 days). All patients remain disease-free at median follow-up of 19 months (range, 12–30 months).

**Conclusion:**

Our experience continues to evolve, and suggests that robotic surgery is safe, feasible and oncologically effective for resection of adrenal masses in well-selected pediatric patients.

## Background

While no randomized trials have evaluated the efficacy of open versus minimally-invasive surgery for treatment of solid abdominal tumors in pediatric populations, the minimally-invasive approach to adrenalectomy is a feasible alternative in well-selected patients [1, 2]. It offers the advantages of improved exposure, reduced soft tissue dissection, improved cosmesis, decreased morbidity and post-operative pain allowing for early feeding, faster return to activity, and decreased length of stay. In adults, it has been associated with decreased blood loss and need for transfusion [3]. Laparoscopic adrenalectomy is the most extensively described minimally-invasive approach, but can be technically challenging due to the small intra-abdominal spaces of pediatric patients, as well as limited instrument dexterity [4].

Use of robotic assistance allows the added advantages of magnified three-dimensional view and articulating instruments with increased range of motion, tremor control, which facilitate precise dissection and hemostasis. Robotic surgery has also been suggested to further reduce duration of hospital stay and blood loss compared to laparoscopic adrenalectomy [5]. However, the body of literature on robotic adrenalectomy for pediatric patients has been sparse, with only two cases reported thus far [6, 7]. We present our technique and initial institutional experience with pediatric adrenalectomies performed using the da Vinci Xi system (Intuitive Surgical, Sunnyvale, CA).

## Case presentation

### Case 1

**Fig. 1:**
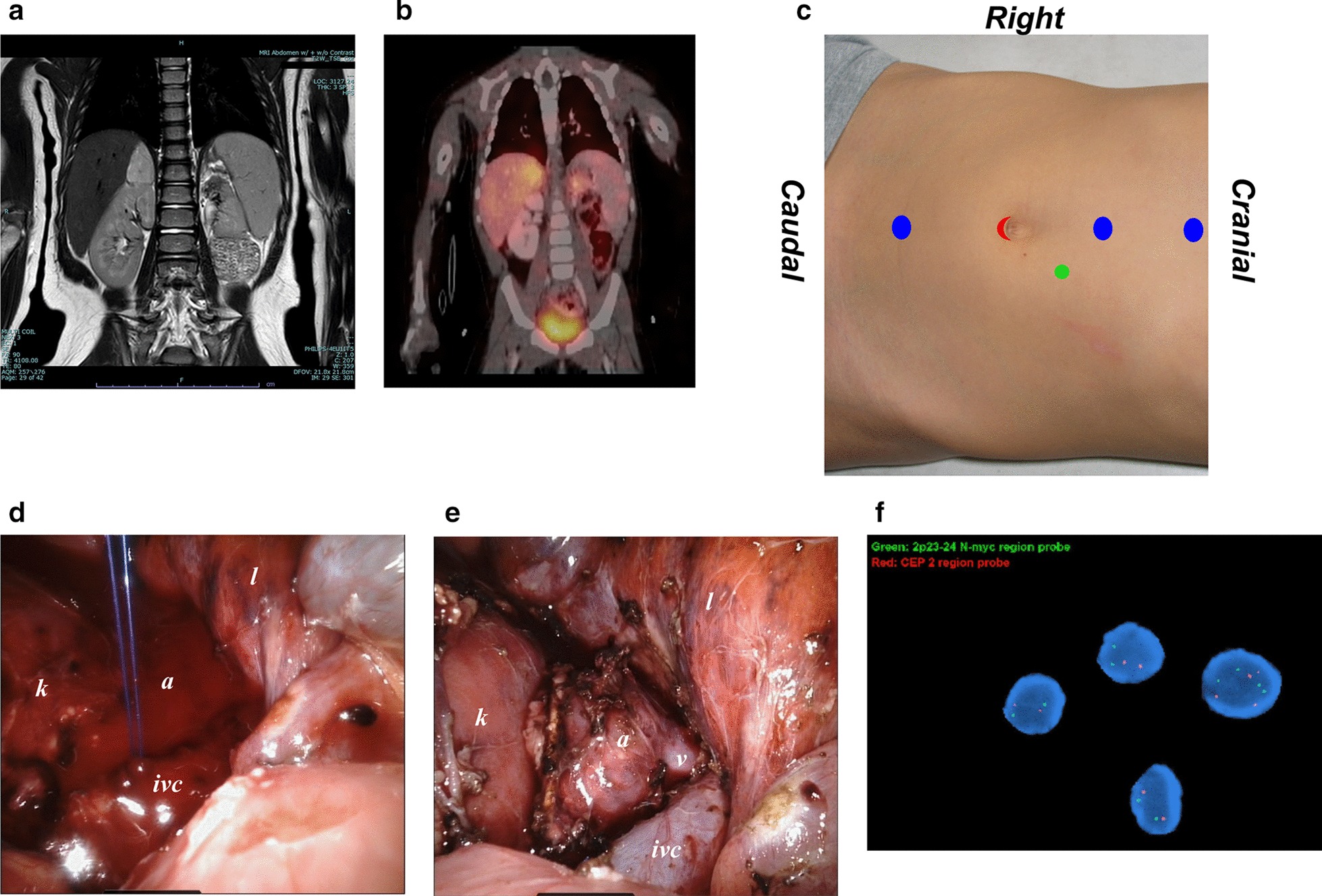
**a** MRI for *Case 1* showing a 2.1 cm × 1.7 cm × 2.5 cm T2 hyperintense, heterogeneously enhancing lobulated structure replacing the right adrenal gland without gross evidence of local invasion and local or distant adenopathy. **b** MIBG study showing activity in the right adrenal gland without any abnormal foci of uptake elsewhere. **c** Location of ports. After placement of an 8 mm periumbilical robotic camera port (red), additional 8 mm midline robotic ports were placed in the suprapubic and subxiphoid regions, and midway between xiphoid and umbilicus (blue) in a straight line. A 5 mm laparoscopic assistant port with insufflation was placed over the right abdominal wall (*green*). Representative intraoperative screen captures for *Case 1* showing **d** primary repair of a small cavotomy with figure-of-eight 4–0 polypropylene suture, and **e** surgical anatomy of the dissected right adrenal gland (*a*) in relation to the right suprarenal vein (*v*), inferior vena cava (*ivc*), right kidney (*k*) and liver (*l*). **f** FISH of the final tumor specimen did not demonstrate *MYCN* amplification

A 2-year-old female had previously undergone a robot-assisted adrenal-sparing left upper pole partial nephrectomy at 14 months of age for recurrent febrile urinary tract infections and a poorly functioning renal moiety with ectopic ureter. Postoperative ultrasound showed a new 2.5 cm right adrenal mass, which was confirmed on MRI. Metabolic activity was confirmed by MIBG study (Fig. 1a, b). 24-h urine metanephrines, homovanillate and vanillylmandelate, and corresponding plasma chemistry levels were within normal limits.

Under general anesthesia, the patient was positioned in left lateral decubitus. An 8 mm robotic camera port was introduced at the umbilicus using the Hasson technique [8]. Following low-pressure insufflation, the camera was introduced and visceral injuries were ruled out. Three additional 8 mm robotic ports and a 5 mm assistant port were then placed (Fig. 1c). After docking the da Vinci Xi robot, the hepatic flexure of the right colon was minimally mobilized to aid in visualization of the right adrenal gland. After identifying the right suprarenal vein and adrenal gland, dissection was carried cephalad under the liver. Combination of monopolar and bipolar cautery was used to obtain hemostasis. A tough dissection plane between the medial aspect of the adrenal gland and the inferior vena cava resulted in creation of a small inadvertent cavotomy that was rapidly repaired primarily with figure-of-eight 4–0 polypropylene suture (Fig. 1d). The right adrenal gland was mobilized (Fig. 1e). The adrenal vein was suture-ligated with 4–0 polypropylene suture before transection. Hemostasis was confirmed and the kidney was well perfused throughout. Specimen was delivered intact through the umbilical port incision using an EndoCatch specimen pouch (Covidien, Minneapolis, MN). Estimated blood loss was 175 ml; 125 ml packed red blood cells, 1000 ml crystalloids and 60 ml colloids were transfused intraoperatively. Operative time was 244 min.

Patient had an uneventful hospital course with good in-house pain control and was discharged on post-operative day two. Final pathological evaluation of the 7.1 gm specimen revealed nodular ganglioneuroblastoma that was negative for c-Myc by immunohistochemistry. FISH did not show *MYCN* amplification (Fig. 1e). Surgical margins were negative. No lymphovascular invasion was noted. Patient is being followed with serial MRI, MIBG scan, and urine catecholamines. She remains disease-free at 30 months of post-operative follow-up.

### Case 2

**Fig. 2:**
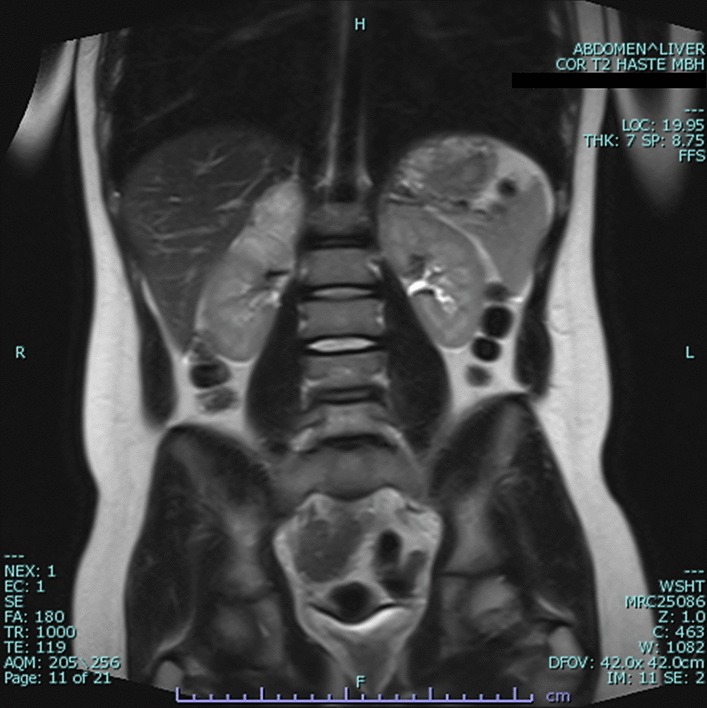
MRI for *Case 2* showing a 6.0 cm × 3.6 cm × 3.2 cm solid enhancing structure in the right adrenal gland region suggestive of a pheochromocytoma

A 13-year-old male was noted to be intermittently tachycardic and hypertensive during hospitalization for a pneumonia episode, and found to have a 6 cm right adrenal mass on MRI (Fig. 2). Patient’s father has von Hippel-Lindau disease and had undergone bilateral adrenalectomy for pheochromocytoma and bilateral nephrectomy for renal cell carcinoma. A pathogenic *VHL* mutation (c.583C > T, p.Q195X) was found in the patient, which was same as the father. 24-h urine norepinephrine level was elevated.

Three weeks after initiating alpha-adrenergic blockade, patient underwent robotic right adrenalectomy. Patient and robotic port positioning were the same as in *Case 1*. Dissection was performed in the plane between the inferior vena cava and the right adrenal gland, and the latter was also released from the right upper renal pole and liver. After freeing the adrenal gland circumferentially without complication, it was delivered intact by extending the suprapubic port incision using an EndoCatch pouch. Estimated blood loss was 100 ml; 2500 ml crystalloids and 750 ml colloids were transfused intraoperatively. Operative time was 244 min.

Patient’s postoperative stay was prolonged due to a generalized morbilliform eruption attributed to a previously unknown allergy to clindamycin. This gradually resolved after administration of systemic corticosteroids and stopping the offending drug, and he was discharged on post-operative day six. Pathological evaluation of the 48.5 gm specimen was consistent with pheochromocytoma. No capsular or vascular invasion was seen. Surgical margins were negative. Patient is being monitored with serial ultrasound and MRI. He remains disease-free at 19 months of post-operative follow-up.

### Case 3

**Fig. 3:**
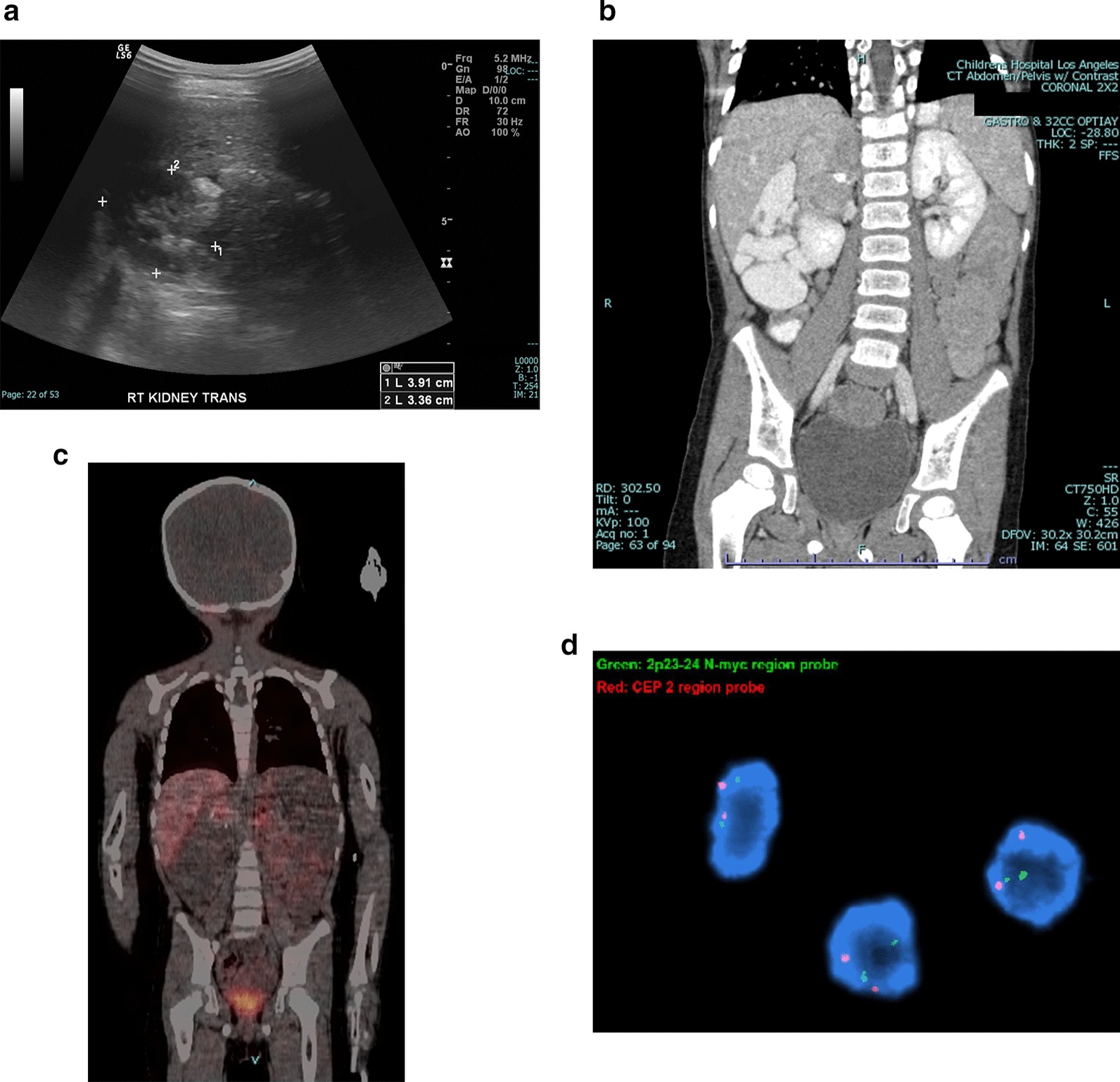
**a** Renal ultrasound for *Case 3* showing a heterogeneous complex mass in the area of the right kidney. **b** CT scan of abdomen and pelvis showing a 6.4 cm × 4.2 cm × 3.4 cm heterogeneous calcified mass replacing the right adrenal gland concerning for neuroblastoma. No regional or distant lymphadenopathy was noted. **c** MIBG study showing activity in the right adrenal gland without evidence of avid metastatic disease. **d** FISH of the final tumor did not demonstrate *MYCN* amplification

Renal ultrasound for workup of microscopic hematuria in a 4-year-old male showed an incidental mass in the area of the right kidney. A subsequent CT scan showed a 6.4 cm heterogeneous mass replacing the right adrenal gland, which was MIBG-avid (Fig. 3a–c). CT chest and bone scan were negative for metastatic disease. 24-h urine homovanillate and vanillylmandelate were elevated.

The patient underwent robotic right adrenalectomy as described in *Case 1*. Dissection was carried over the inferior vena cava, and small vessels feeding into the mass were controlled with bipolar electrocautery before transection. A prominent lymph node overlying the vena cava was also excised. Specimen was delivered through a mini Pfannenstiel incision along the suprapubic port. Estimated blood loss was 15 ml; 500 ml crystalloids were transfused intraoperatively. Operative time was 265 min.

Patient had an uneventful hospital course and was discharged on post-operative day one. Pathological diagnosis of the 57 gm specimen was intermixed type, Schwannian stroma-rich ganglioneuroblastoma with negative surgical margins. Immunohistochemistry was negative for c-Myc and n-Myc. FISH did not show *MYCN* amplification (Fig. 3d). No malignancy was identified in the resected lymph node. Patient is being followed with serial MRI, MIBG scan, and urine catecholamines. He remains disease-free at 12 months of post-operative follow-up.

## Discussion and conclusions

We herein report on the initial experience with robotic pediatric adrenalectomy from our institution. All cases followed a standardized surgical approach independent of suspected adrenal pathology, and none required open conversion. All specimens were resected and delivered intact (median weight, 48.5 g; range, 7.1–57 g), and all surgical margins were negative.

We demonstrate that robotic adrenalectomy is feasible in a wide range of ages and body habitus in the pediatric population. While use of robotic assistance has been described in two pediatric adrenalectomy cases previously [6, 7], this is the first reported series using the da Vinci Xi robot. This provides additional advantages over prior generation robots including improved patient arm clearance, versatility of camera placement into any port, ability to place arms along a single line thereby avoiding clashing, and guided docking and targeting [9].

Median operative time was 244 min (range, 244–265 min), and reflects our initial learning curve in performing this complex surgery at a tertiary center with more than 50% trainee participation in each case. As with most complex robotic procedures, we do anticipate that operative time will continue to improve as our experience matures. However, one is cautioned by findings from the adult population that indicate significantly longer operating time in patients who underwent robotic adrenalectomy when compared with those undergoing laparoscopic resection [10]. Median estimated blood loss was 100 ml (range, 15–175 ml). Injury to surrounding vasculature is a known complication during adrenalectomy; *Case 1* also demonstrates ability of the da Vinci Xi system for rapid control and primary repair of such injuries due to precise articulation of the robotic instruments.

While median post-operative hospital stay for this series was 2 days, one patient had a protracted recovery due to an unexpected drug reaction (range, 1–6 days). Typical postoperative recovery, however, appears to be similar or better to prior reports for minimally-invasive adrenalectomies [4, 6, 7]. At a median post-operative follow-up of 19 months (range, 12–30 months), all patients remain disease-free. Our series represents the longest reported follow-up for pediatric patients undergoing robotic adrenalectomy. There were no mortality and local recurrence or metastases, and biochemical and hormonal parameters have remained within normal limits for functional tumors in this series. This further demonstrates the oncologic safety and efficacy of this approach.

In conclusion, robotic assistance has been successfully and reproducibly used for resecting adrenal masses in adults. We report the first series of pediatric adrenalectomy using the latest generation da Vinci Xi robot. Our experience demonstrates that robot-assisted adrenalectomy is a safe, effective and viable option in well-selected pediatric patients. Surgical excision can be accomplished with rapid recovery time, relatively low surgical morbidity, and comparable oncologic outcomes. While adhering to sound oncologic principles, a robotic approach provides added advantages of improved visualization, precision and dexterity. Maturing operative experience and larger series may be able to address if this approach can potentially improve surgical outcomes.

## Data Availability

All data supporting our findings and conclusions are contained within the manuscript, and any missing details will be shared upon request.

## Abbreviations

MRI: Magnetic resonance imaging
MIBG: Metaiodobenzylguanidine
FISH: Fluorescent in situ hybridization
CT: Computed tomography
